# Cancer-related self-perception in men affected by prostate cancer after radical prostatectomy

**DOI:** 10.1007/s11764-022-01256-2

**Published:** 2022-09-13

**Authors:** Matthias Jahnen, Luisa Lehner, Valentin H. Meissner, Andreas Dinkel, Stefan Schiele, Helga Schulwitz, Jürgen E. Gschwend, Kathleen Herkommer

**Affiliations:** 1 Department of Urology, Klinikum rechts der Isar, School of Medicine, Technical University of Munich, Ismaninger Str. 22, 81675 Munich, Germany; 2 Department of Psychosomatic Medicine and Psychotherapy, Klinikum rechts der Isar, School of Medicine, Technical University of Munich, Langerstr.3, 81675 Munich, Germany

**Keywords:** Prostate cancer, Cancer-related identity, Self-perception, Cancer survivors, Survivorship, Radical prostatectomy

## Abstract

**Purpose:**

To identify factors associated with cancer-related self-perception after being affected by prostate cancer (PCa) and radical prostatectomy.

**Subjects and methods:**

Men affected by PCa and radical prostatectomy were asked to choose one of 5 cancer-related identities (“patient,” “victim,” “someone who has had cancer,” “cancer survivor,” and “cancer conqueror”). Associations with clinical data, functional outcome (continence and sexual activity), and psychological factors were assessed.

**Results:**

One thousand seven hundred seventy-two men were included. Most men perceived themselves as “someone who has had cancer” (46.8%) which was associated with no cancer recurrence (OR: 0.54 [0.36–0.81]) and low cancer-related distress (OR: 0.69 [0.53–0.89]) or “patient” (35.4%) which was associated with ongoing therapy (OR: 2.59 [1.59–4.22]) and biochemical disease recurrence (OR: 1.91 [1.28–2.85]). Self-perception, as “cancer survivor” (7.8%), “cancer conqueror” (8.2%), or “victim” (1.8%), was less common. “Cancer survivor” was associated with high perceived disease severity (OR: 2.07 [1.33–3.24]) and incontinence (1.99 [1.27–3.12]). “Cancer survivor” and “cancer conqueror” were related to high benefit finding (OR: 2.05 [1.30–3.23], OR: 1.89 [1.27–2.81], respectively); only “cancer conqueror” was associated with higher quality of life (OR: 1.38 [1.21–1.58]).

**Conclusions:**

Self-perception in men affected by PCa can vary widely and is associated with distinct characteristics that reflect the experienced severity of the disease, therapy side effects, and psychological well-being.

**Implications for Cancer Survivors:**

The assessment of cancer-related self-perception can give important insights when evaluating men affected by PCa who need assistance in coping with their disease.

## Background

With an aging population along with improvements in early detection, there are approximately 450,000 men diagnosed with prostate cancer (PCa) in Europe annually [12]. In most men, PCa is diagnosed in an early, symptomless stage and can be treated successfully, guaranteeing long-term survival [9]. However, living with a history of PCa beyond primary therapy is in many cases still accompanied by a wide range of hardships deriving from factors such as distrust in one’s body and therapy side effects, most significantly incontinence and erectile dysfunction [7, 25]. Further, an ongoing cancer follow-up may cause lingering existential fears of a tumor progression leading to a symptomatic metastasized disease [21]. Taken together, this has placed growing importance on advising men affected by PCa on how to process their cancer experience and how to integrate it into their personal self-perception.

In this regard, it has been proposed that the adaptation of an active cancer-related identity, which accentuates overcoming the disease, might help coping with cancer and its therapy side effects [10, 24]. Particularly in the USA, the concept of survivorship and identification as a “cancer survivor” has been advocated for individuals affected by cancer in order to encourage such a way of self-perception [8, 19]. Furthermore, it has been proposed to abandon terminology such as “patient” or “cancer victim,” which connotate a more passive stance and are regarded as contrary towards developing a positive self-perception when being affected by cancer [2, 10, 20].

In several studies on individuals affected by diverse types of cancer, self-identification as “cancer survivor” has been associated with more active disease coping, higher participation in cancer-related activities, and better psychological well-being[2, 8]. However, subsequent research has revealed that the majority of individuals affected by cancer rather identify with more neutral terms such as “someone who has had cancer” without a major drawback in overall well-being [8]. Regarding men affected by PCa, small US studies showed that a third of men affected by PCa favor terms such as “cancer survivor” or “cancer conqueror” as self-description [2, 23]. In these studies, identification as a “survivor” was associated with positive affect, and it has been suggested that adoption of a “survivor” identity might be associated with lower threat appraisal, thoughtful reflection, and gaining an understanding through peers [2, 23, 24]. This research implies that despite the in general good long-term survival prognosis, cancer-related self-perception might influence psychological adaptation and overall well-being in men affected by PCa. In a recent study on men affected by PCa of a Germany wide research project with a median follow up of more than 15 years, we were already able to show that differences in cancer-related self-perception are measurable even years after primary therapy and that different cancer-related identities are associated with specific psychological factors. Further, we were able to show that some clinical factors such as cancer recurrence and ongoing therapy were associated with cancer-related self-perception, but that also the subjective experienced severity of the disease remained an independent major factor associated with a certain cancer-related self-perception. We found a positive association between high perceived disease severity and self-identification as “survivor” or “victim” as well as an association between low perceived disease severity and self-identification with the less loaded term “someone who has had cancer”[14]. The subjective disease burden is often not only a reflection of the disease itself but of the consequences and side effects from the necessary therapy. Radical prostatectomy is often associated with therapy side effects such as incontinence and erectile dysfunction which influence men affected by PCa physically and psychologically especially in the first few years after radical prostatectomy. However, in which way, the development of a particular form of cancer-related self-perception is associated with therapy side effects, and specific cancer-related distress has so far not been investigated.

This study addresses these remaining issues regarding cancer-related self-perception in men affected by PCa by analyzing a large sample of German men 1 to 12 years after radical prostatectomy. First, it was assessed how these men self-identify with the following 5 cancer-related identities: “patient,” “victim,” “someone who has had cancer,” “cancer survivor,” and “cancer conqueror.” Second, differences in detailed clinical data, functional outcome (continence and sexual activity), and psychological characteristic, reflecting the subjective cancer experience and its psychological impact, between each cancer-related self-perception were examined. Lastly, 5 separate multivariable models were calculated to identify independent associations between cancer-related self-perception and the analyzed factors, in order to demonstrate that differences in self-perception after radical prostatectomy may have clinically significant implications for men affected by PCa.

## Subjects and methods

### Study sample

Since 2006, men treated for PCa with radical prostatectomy at the *Department of Urology of the Klinikum rechts der Isar, Technical University of Munich*, are asked prior to surgery to participate in psycho-oncological research projects and the hospitals PCa register, containing standardized sociodemographic, clinical, and functional data of all treated PCa patients. These men are contacted annually via mail to complete ongoing questionnaires concerning current clinical, functional, sociodemographic, and psychosocial information. Further clinical and pre-surgical information for this analysis was obtained through the clinics PCa register. All participants gave their written consent to participate. The ethics committee of the Technical University of Munich has approved this research project.

For this cross-sectional analysis, men were contacted via mail between November 2019 and October 2020. Men who underwent primary radical prostatectomy (without neoadjuvant therapy) between one and 12 years prior to survey and who answered the item regarding cancer-related identity were included (*n* = 1772).

## Measures

### Cancer-related self-perception

Participants were asked to choose one of the following terms that would describe them most suitable with regards to their cancer experience [2, 8, 10]: “patient,” “victim,” “someone who has had cancer,” “cancer survivor,” and “cancer conqueror.”

### Sociodemographic and clinical characteristics

The following sociodemographic data were included in this analysis: age at survey, current partnership, and children. Clinical data included were age at surgery, time since surgery, presence of a second primary cancer, family history of PCa (yes: at least one consanguine relative with PCa vs no), family history of cancer (other than PCa), PSA level at diagnosis, histopathological Gleason grade group, organ-confined stage at RP according to TNM classification of 2002, biochemical recurrence (PSA level ≥ 0.2 ng/ml) during follow-up (at any time during follow-up vs ongoing at survey vs no), ongoing PCa treatment at survey, adjuvant therapy (radiotherapy (+ / − androgen deprivation therapy) vs androgen deprivation therapy vs no), and Royal College of Surgeons Charlson comorbidity index before surgery [6].

### Functional outcome

The following data on sexual and bladder function were assessed prior to surgery and post-surgery (at survey): urinary continence (urinary continence was defined prior to surgery in accordance to the International Consultation on Incontinence Questionnaire Short-Form (ICIQ-SF). A sum score > 5 was considered as incontinence [1]. Incontinence post-surgery was defined in accordance with the 24-h pad test. The reporting of ≥ 1 wet pad within 24 h was considered as incontinence [13]); masturbation (in the last 4 weeks); and partnered sexual activity (any form of sexual activity performed with a partner) (in the last 4 weeks). Masturbation and partnered sexual activity were further combined into a single variable (sexual activity independent of partner) in order to represent any form of sexual activity in the last 4 weeks.

### Depression and anxiety

Symptoms of depression and anxiety were assessed using the validated ultra-brief instruments Patient Health Questionnaire-2 (PHQ-2) and General Anxiety Disorder-2 (GAD-2) scale. For both scales (range 0–6), a cut-off score ≥ 3 indicates a positive screening of depression or anxiety, respectively [17, 18].

### Distress and psychosocial counseling

Distress was assessed with the short form of the questionnaire on distress in cancer patients (QSC-R10) using 10 items that capture cancer specific stressors. Participants were asked to answer on a six-point scale ranging from “applies and hardly distresses me” [1] to “applies and distresses me severely” [5] (not applicable [0]). A sum score > 14 was used as an indicator for high distress. Following the QSC-R10, all men were asked whether they desire psychosocial counseling [5].

### Global health status/quality of life

Quality of life was assessed using the last 2 items of the European Organization for Research and Treatment of Cancer questionnaire (EORTC QLQ-C30). These two items capture the overall health and quality of life in the past week. Participants were asked to answer on a seven-point Likert scale ranging from “very poor” [1] to “excellent” [7]. Based on the standardized EORTC formula, the mean value of the two items was calculated to a score, ranging from 0 to 100. Higher scores indicate a higher quality of life [11].

### Perceived severity of the disease

The perceived severity of being affected by PCa was assessed with the single item “Having had prostate cancer is one of the worst things that happened to me in my life” (adapted from [30]). Participants were asked to answer on a four-point Likert scale ranging from “strongly disagree” [1] to “strongly agree” [4]. Responses (1) and (2) as well as responses [3] and [4] were combined to “low perceived severity” and “high perceived severity,” respectively.

### Benefit finding

Benefit finding was assessed using one item with high factor loading and high face validity adapted from the German version of the 17-item benefit finding scale: “My prostate cancer has helped me become more focused on priorities, with a deeper sense of purpose in life” [22]. Participants were asked to answer on a five-point Likert scale ranging from “not at all” [1] to “extremely” [5]. Responses [1] and [2] as well as [3] to [5] were combined to “low benefit finding” and “high benefit finding,” respectively.

### Statistical analysis

Descriptive statistics were calculated for all study variables. Chi-square and Wilcoxon tests were applied for analyzing differences in cancer-related self-perception with regard to sociodemographic, clinical, and psychological variables. To identify variables independently associated with each of the 4 different ways of cancer-related self-perception multivariable logistic regression with backward elimination was used. Significance was set at *p* < 0.05. All analyses were performed using SAS (Version 9.4).

## Results

**Fig. 1:**
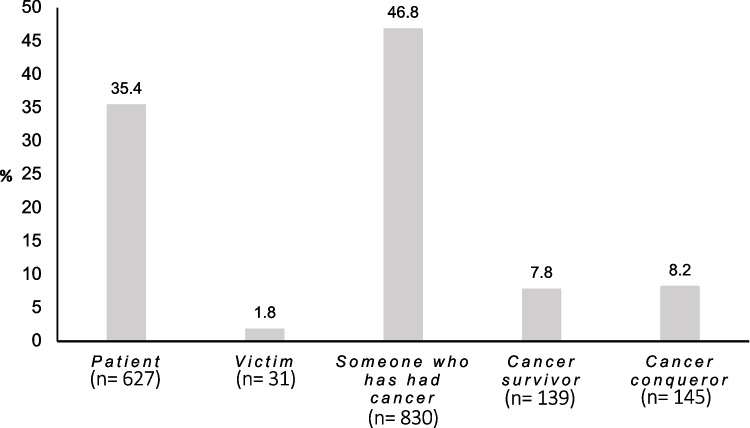
Self-identification in the study sample of men affected by prostate cancer with a median follow-up of 4 years

One thousand seven hundred seventy-two men affected by PCa and primarily treated with radical prostatectomy with a mean age of 70.9 years at survey (standard deviation (SD) = 8.0) and a median follow-up of 4 years (first and 3rd quartile = 2–8) were included in the analysis (Table 1). Men self-identified most frequently as “someone who has had cancer” (46.8%) followed by “patient” (35.4%). The terms “cancer conqueror” and “cancer survivor” were favored by 8.2% and 7.8%, respectively. The least endorsed term was “victim” (1.8%) (Fig. 1).

**Table 1:** Sociodemographic, clinical, and psychological characteristics of the study sample (*n* = 1772)

			*n*	%
Cancer-related self-perception				
Patient			627	35.4
Victim			31	1.8
Someone who has had cancer			830	46.8
Cancer survivor			139	7.8
Cancer conqueror			145	8.2
Sociodemographic characteristics				
Age at survey (years)	M: 70.9 SD: 8.0			
≤ 60			182	10.3
> 60 to ≤ 70			568	32.0
70			1022	57.7
Partnership				
Yes			1438	86.7
No			220	13.3
Children				
0			294	19.2
≥ 1			1237	80.8
Clinical characteristics				
Age at surgery (years)	M: 65.8 SD: 7.7			
≤ 55			167	9.4
> 55 to ≤ 65			592	33.4
> 65			1013	57.2
Time since surgery (years)	Mdn: 4 [2−8]			
1–2			491	27.7
3–5			570	32.2
> 5			711	40.1
Second primary cancer				
Yes			143	8.1
No			1629	91.9
Family history of PCa				
Yes			495	27.9
No			1277	72.1
Family history of cancer (other than PCa)				
Yes			796	44.9
No			976	55.1
PSA level at diagnosis (ng/ml)	Mdn: 7.0 [5.1–10.6]			
≤ 4			178	10.1
> 4 to ≤ 10			1110	62.6
> 10			484	27.3
Gleason grade groups (1–5)				
ISUP 1			255	14.3
ISUP 2			792	44.8
ISUP 3			447	25.3
ISUP 4			100	5.7
ISUP 5			176	9.9
Organ-confined stage at RP				
Yes			1041	58.8
No			731	41.2
Biochemical recurrence				
Yes (biochemical recurrence ongoing)			180	10.3
Yes (biochemical recurrence not ongoing)			211	12.1
No			1352	77.6
Ongoing treatment at survey				
Yes			139	7.8
No			1633	92.2
Adjuvant therapy				
Radiotherapy (+ / − androgen deprivation therapy)			165	9.3
Androgen deprivation therapy			66	3.7
No			1541	87.0
RCS Charlson Score (at surgery)				
0			1509	85.2
1			204	11.5
≥ 2			59	3.3
Functional outcome				
Continence				
Yes			1309	75.5
No (continent prior to surgery)			426	24.6
No (not continent prior to surgery)			11	0.6
Overall sexual activity independent of partner				
Yes			1090	70.5
No (sexual activity prior to surgery)			327	21.1
No (no sexual activity prior to surgery)			130	8.4
Masturbation (in the last 4 weeks)				
Yes			871	55.3
No (masturbation prior to surgery)			108	6.9
No (no masturbation prior to surgery)			239	15.2
No (no presurgical information)			356	22.6
Partnered sexual activity (in the last 4 weeks)				
Yes			638	42.6
No (partnered sexual activity prior to surgery)			512	34.2
No (no partnered sexual activity prior to surgery)			347	23.2
Psychosocial factors				
PHQ-2 (depression screening)				
Positive screening (≥ 3)			140	8.0
Negative screening (< 3)			1601	92.0
GAD-2 (anxiety disorder screening)				
Positive screening (≥ 3)			130	7.5
Negative screening (< 3)			1595	92.5
Distress (QSC-R10) FBK10		Mdn: 7 [3−14]		
High (> 14)			397	23.5
Low (≤ 14)			1291	76.5
Psychosocial counseling desired				
Yes			410	23.8
No			1314	76.2
Quality of life EORTC QLQ-C30		M: 73.7 SD: 18.5		
Perceived severity of disease				
High			780	44.7
Low			965	55.3
Benefit finding				
High			861	50.0
Low			861	50.0

*M*, mean; *SD,* standard deviation; *Mdn*, median; *PCa,* prostate cancer; *PSA,* prostate specific antigen; ISUP, The International Society of Urological Pathology; *RP,* radical prostatectomy; *RCS*, Royal College of Surgeons*; PHQ,* patient health questionnaire; *GAD,* general anxiety disorder; *QSC-R10,* Questionnaire on Stress in Cancer Patients*; EORTC QLQ*, European Organization for Research and Treatment of Cancer Quality of Life Questionnaire

Men who self-identified as “someone who has had cancer” were the youngest at surgery, most often sexually active (76.6% vs. 64.1% (rest of the sample)) and had the lowest percentage of biochemical recurrence during follow-up (15.0% vs. 28.8% (rest of the sample)). Incontinence at survey was most often reported by men who self-identified as “cancer survivor” or “victim” (38.0% and 60.0%, respectively, vs 23.2% (rest of the sample)). These men reported also most often high perceived severity of disease (61.5% and 90.3%, respectively, vs. 42.6% (rest of the sample)) and most often high cancer-related distress (35.8% and 58.1%, respectively, vs. 21.9% (rest of the sample)). While men who self-identified as “cancer conqueror” expressed the highest quality of life (QLQ-C30: 81.0 ± 15.4), men who self-identified as “cancer survivor” or “victim” expressed lowest quality of life (QLQ-C30: 69.1 ± 20.3 and QLQ-C30: 54.3 ± 18.6, respectively). High benefit finding was found most often in men self-identified as “cancer survivor” or “cancer conqueror” (68.9% and 65.3%, respectively, vs. 46.7% rest of the sample)) (all *p* < 0.0001, Table 1, Table 2).

**Table 2:** Comparison of key characteristics of the 5 cancer-related identities

		Someone who has had cancer *n* = 830 (%)	Patient *n* = 627 (%)	Cancer conqueror *n* = 145 (%)	Cancer survivor *n* = 139 (%)	Victim *n* = 31 (%)
Age at survey (years)	***p* = *.0008***					
≤ 60		13.0	8.6	6.9	5.8	6.5
> 60 to ≤ 70		35.1	28.7	32.4	30.2	25.8
> 70		51.9	62.7	60.7	64.0	67.7
Partnership	*p* = *.44*					
Yes		85.8	86.4	91.3	88.2	90.3
No		14.2	13.6	8.7	11.8	9.7
Children	*p* = *.22*					
≥ 1		80.7	79.7	78.0	88.4	84.0
0		19.3	20.3	22.0	11.6	16.0
Age at surgery (years)	***p* < *.0001***					
≤ 55		11.5	8.8	8.3	2.2	6.5
> 55 to ≤ 65		37.7	27.6	32.4	36.7	25.8
> 65		50.8	63.6	59.3	61.1	67.7
Time since surgery (years)	*p* = *.12*					
1–2		29.8	27.9	22.1	18.7	35.5
3–5		31.5	33.0	34.5	31.7	25.8
> 5		38.7	39.1	43.4	49.6	38.7
Second primary cancer	*p* = *.93*					
Yes		8.7	7.5	7.6	7.9	6.5
No		91.3	92.5	92.4	92.1	93.5
Family history of PCa	*p* = *.43*					
Yes		28.4	27.6	29.7	28.1	12.9
No		71.6	72.4	70.3	71.9	87.1
Family history of cancer (other than PCa)	*p* = *.056*					
Yes		46.8	42.4	46.2	39.6	64.5
No		53.2	57.6	53.8	60.4	35.5
PSA Lever at diagnosis (ng/ml)	***p* = *.006***					
≤ 4		11.9	8.3	8.3	9.4	6.5
> 4 ≤ 10		65.3	61.6	56.5	57.5	64.5
> 10		22.8	30.1	35.2	33.1	29.0
Gleason grade group (1–5)	*p* < *.0001*					
ISUP 1		17.7	11.7	14.5	10.1	0.0
ISUP 2		48.7	42.3	40.0	40.3	29.0
ISUP 3		22.6	27.8	29.0	21.6	45.2
ISUP 4		4.1	6.1	9.7	8.6	6.5
ISUP 5		6.9	12.1	6.9	19.4	19.4
Organ-confined stage at RP	***p* < *.0001***					
Yes		65.7	54.6	57.9	41.0	41.9
No		34.3	45.5	42.1	59.0	58.1
Biochemical recurrence	***p* < *.0001***					
Yes (progress not ongoing)		9.0	15.5	15.4	10.1	20.7
Yes (progress ongoing)		6.0	14.9	7.0	18.1	17.2
No		85.0	69.8	77.6	71.7	62.1
Ongoing treatment at survey	***p* < *.0001***					
Yes		3.1	13.6	2.1	13.7	19.4
No		96.9	86.4	97.9	86.3	80.7
Adjuvant therapy	***p* < *.0001***					
Radiotherapy		6.6	9.7	12.4	18.0	19.4
Androgen deprivation therapy		2.2	5.3	4.1	5.8	3.2
No		91.2	85.0	83.5	76.2	77.4
RCS Charlson Score (at surgery)	***p* = *.015***					
0		88.0	83.6	82.1	79.8	77.4
1		10.1	12.0	11.0	17.3	16.2
≥ 2		1.9	4.4	6.9	2.9	6.4
Urinary continence	***p* < *.0001***					
Yes		77.0	77.5	78.2	62.0	40.0
No (continent prior to surgery)		23.0	22.5	21.8	38.0	60.0
Sexual activity independent of partner	***p* = *.0002***					
Yes		76.6	65.7	64.1	61.5	59.3
No (sexual activity prior to surgery)		17.1	24.1	27.5	25.7	26.0
No (no sexual activity prior to surgery)		6.3	10.2	8.4	12.9	14.8
Masturbation	***p* < *.0001***					
Yes		63.6	49.4	50.4	41.7	32.1
No (masturbation prior to surgery)		6.4	7.3	8.5	5.8	7.1
No (no masturbation prior to surgery)		12.4	16.6	16.3	22.5	25.0
No		17.6	26.7	24.8	30.0	35.7
Partnered sexual activity	***p* = *.042***					
Yes		46.5	40.5	38.9	34.3	30.8
No (partnered sexual activity prior to surgery)		33.5	34.6	36.5	32.4	42.3
No (no partnered sexual activity prior)		20.0	25.0	24.6	33.3	26.9
PHQ-2 (depression screening)	***p* < *.0001***					
Positive screening (≥ 3)		6.4	8.9	5.6	12.7	27.6
Negative screening (< 3)		93.6	91.1	94.4	87.3	72.4
GAD-2 (anxiety disorder screening)	***p* < *.0001***					
Positive screening (≥ 3)		6.1	7.3	4.9	15.3	29.0
Negative screening (< 3)		93.9	92.7	95.1	84.7	71.0
Quality of life QLQ-C30	***p* < *.0001***	MW: 74.7 SD:17.7	MW: 72.7 SD:18.9	MW: 81.0 SD:15.4	MW: 69.1 SD:20.3	MW: 54.3 SD:18.6
Distress (QSC-R10) FBK10	***p* < *.0001***					
High (> 14)		19.4	26.4	14.7	35.8	58.1
Low (≤ 14)		80.6	73.6	85.3	64.2	41.9
Psychosocial counseling desired*	***p* = *.018***					
Yes		21.6	26.3	17.5	29.9	34.5
No		78.4	73.7	82.5	70.1	65.5
Perceived severity of disease	***p* < *.0001***					
High		42.0	42.9	42.7	61.5	90.3
Low		58.0	57.1	57.3	38.5	9.7
Benefit finding	***p* < *.0001***					
High		48.1	44.7	65.3	68.9	51.6
Low		51.9	55.3	34.7	31.1	48.4

*PCa,* prostate cancer; *PSA,* prostate specific antigen; ISUP, The International Society of Urological Pathology; *RP,* radical prostatectomy; *RCS*, Royal College of Surgeons*; PHQ,* patient health questionnaire; *GAD,* general anxiety disorder; *QSC-R10,* Questionnaire on Stress in Cancer Patients*; EORTC QLQ*, European Organization for Research and Treatment of Cancer Quality of Life Questionnaire

Multivariable logistic regression analyses showed an association between men who self-identified as “someone who has had cancer” and a younger age at survey (OR: 0.98 [0.97–0.99]). Men who experienced a biochemical recurrence (OR: 1.91 [1.28–2.85]) or an ongoing therapy at survey (OR: 2.59 [1.59–4.22]) were more likely to self-identify as “patient,” whereas men who did not experience biochemical recurrence were more likely to self-identify as “someone who has had cancer” (OR: 0.54 [0.36–0.81]). Men who were incontinent at survey were more likely to self-identify as “cancer survivor” 1.99 [1.27–3.12]) and men who were not sexually active in the 4 weeks prior to survey were more likely to self-identify as “cancer conqueror” (OR: 1.83 [1.15–2.91]). No Incontinence at survey was associated with self-identification as “patient” (OR: 0.72 [0.54–0.96]) (all *p* < 0.05, Table 3).

**Table 3:** Factors associated with cancer-related identities in multiple logistic regression analysis with backward elimination

	Someone who has had cancer OR [95% CI]	Patient OR [95% CI]	Cancer conqueror OR [95% CI]	Cancer survivor OR [95% CI]
Age at survey (years) [continues]	0.98 [0.97–0.99]			
Ongoing treatment at survey [ref: no]				
Yes	0.43 [0.25–0.74]	2.59 [1.59–4.22]	0.22 [0.05–0.93]	
Biochemical recurrence [ref: no]				
Yes (progress not ongoing)	0.66 [0.47–0.94]	1.57 [1.10–2.23]		
Yes (progress ongoing)	0.54 [0.36–0.81]	1.91 [1.28–2.85]		
Continence [ref: yes]				
No (continent prior to surgery)		0.72 [0.54–0.96]		1.99 [1.27–3.12]
Sexual activity independent of partner [ref: yes]				
No (sexual activity prior to surgery)			1.83 [1.15–2.91]	
No (no sexual activity prior to surgery)			1.36 [0.65–2.85]	
Quality of life QLQ-C30 [continues]			1.38 [1.21–1.58]	
Distress (QSC-R10) FBK10 [ref: low (≤ 14)]				
High (> 14)	0.69 [0.53–0.89]	1.41 [1.07–1.86]		
Perceived severity of disease [ref: low]				
High				2.07 [1.33–3.24]
Benefit finding [ref: low]				
High		0.66 [0.52–0.83]	1.89 [1.27–2.81]	2.05 [1.30–3.23]

*PCa,* prostate cancer; *PHQ,* patient health questionnaire; *FU*, follow-up; *ref*, reference; *OR*, odds ratio; *CI*, confidence interval; *sec.*, secondary

Whereas men who reported low cancer-related distress were more likely to self-identify as “someone who has had cancer” (OR: 0.69 [0.53–0.89]), men reporting high cancer-related distress were more likely to self-identify as “patient” (OR: 1.41 [1.07–1.86]). High benefit finding was associated with self-identification as “cancer conqueror” or “cancer survivor” finding (OR: 2.05 [1.30–3.23], OR: 1.89 [1.27–2.81], respectively), and higher quality of life was associated with self-identification as “cancer conqueror” (OR: 1.38 [1.21–1.58]). High perceived severity of the disease was associated with self-identification as “cancer survivor” (OR: 2.07 [1.33–3.24]) (Table 3) (all *p* < 0.05). Due to the small sample size (*n* = 31), no multivariable logistic regression model was calculated for men identifying as “victim.”

## Discussion

Life expectancy after diagnosis and treatment for PCa is high due to early detection and effective treatment options [12]. Nevertheless, the psychological weight of a cancer diagnosis, fear of disease progression, and treatment side effects are a psychological burden for many men affected by PCa [7]. Therefore, it is important to learn more about the psychological adaptation of these men.

In this analysis on 1,772 men affected by prostate cancer (PCa) with a median follow-up of 4 years after radical prostatectomy, most men self-identified as “someone who has had cancer” (46.8%) followed by “patient” (35.4%). Less than 20% of men perceived themselves as “cancer conqueror” or “cancer survivor” and only a minority of men reported that “victim” would describe them best (1.8%). These results are in accordance with previous research on men affected by PCa, which has shown that, while the majority of these men identify themselves with neutral term such as “someone who has had cancer,” cancer–related self-perception with regard to one’s personal PCa experience may vary widely [8]. Moreover, compared to the results of our previous study on men affected by PCa with a very long follow-up (median 15.6 years), no major differences in the distribution of cancer-related self-perception can be observed. However, in this previous study, twice as many men (16.8%) self-identified as “survivor” while self-identification with “someone who has had cancer” and “patient” was somewhat lower, suggesting that the adaptation of a “survivor” identity might develop with higher age and years after cancer diagnosis and subsequent therapy. Additionally, by adding data on functional outcome after radical prostatectomy as well as detailed psychological data, reflecting cancer-related distress and psychological adaptation, we were able to further expand the understanding of cancer-related self-perception in men affected by PCa in this study.

In men who self-identified as “someone who has had cancer,” PCa was more often diagnosed in an organ confined stage with fewer oncological risk factors. Consequently, these men were more likely to have experienced no tumor recurrence after primary therapy and were less likely to require adjuvant therapy. Further, these men reported a high postoperative rate of continence (77.0%) and the highest rate of postoperative sexual activity (76.6%), and were less likely to be affected by cancer-related distress. These results show that due to efficient early detection of PCa, enabling primary therapy with excellent oncological and functional outcomes possible, the personal PCa experience in many men does not have the psychological demanding weight to trigger a deeper, more active cancer-related identity [26]. Qualitative studies have revealed that these men perceive their cancer experience as something of the past [24]. Taken together, this suggests that state of the art early detection and treatment options enable PCa therapy that not only guarantees a long overall survival but also a minimal burden on the physiological and psychological quality of life beyond cancer therapy.

One third of the men surveyed in this analysis self-identified as “patient.” These men were more likely to have experienced cancer recurrence or to receive an ongoing PCa therapy. For these men, PCa is a continuing reality rather than an overcome life event. Therefore, it is not surprising that these men were also more likely to report cancer-related distress. However, this distress does not seem to be a consequence of therapy side effects from primary therapy, as these men were also more likely to be continent at survey. Further, most men who identified as patient did not report high perceived disease severity and did not display a deficit in their quality of life compared to the age-matched men from the general German population [27]. This is in contrast to previous studies that have suggested that continued self-identification as “(cancer) patient” after primary therapy might be a sign of submission and passiveness, which might lead to a reduced psychological well-being [10, 29]. Data of our analysis show that the term “patient” might rather be favored as neutral description of an ongoing interaction with a medical condition and its required treatment phases. Such a way of self-perception might be especially common in cultures with clear and direct social traits such as Germany and therefore explain the difference between our data and data from the USA.

In most scientific literature, individuals affected by cancer after primary therapy are in general referred to as “cancer survivors” [28]. The term originates from the commonly used terms “overall survival” or “recurrence free survival,” which describe the time length of survival after diagnosis in order to illustrate the aggressiveness of a certain kind of cancerous disease. Further, 3 or 5 years of recurrence free survival are often considered as time frames, which indicate that individuals affected by cancer might be considered as cured [28]. With growing research on cancer survivorship, the term “cancer survivor” has also been advocated as proper description of individuals affected by cancer regardless of disease course in order to emphasize resilience and personal strength in one’s “fight” against cancer. In support of such a mindset, public cancer survivorship movements have become a part of especially North-American culture [8]. However, research has shown that a large portion of individuals affected by cancer does not identify as much with the term, especially when lacking a socio-cultural support background, that has branded the term as something empowering [16, 28]. Additionally, men affected by PCa have stated in previous studies that they did not experience PCa as such a threatening event to be reflected in the term “survivor”[4, 16, 21]. The data of this analysis reflects this. Identification with the term “survivor” was lower than in studies from the USA, showing that outside of the US identification with the term is less popular and might be interpreted differently [2, 8]. Men who endorsed the term “survivor” were more likely to have a high perceived disease severity and reported more often fundamental life changes (benefit finding) due to their cancer experience reflecting the idea that in order to feel like a “survivor,” one has to overcome a life event with a certain gravity. Moreover, these men were more likely to be incontinent at survey. Incontinence is one of the most psychological demanding side effects of radical prostatectomy and may have a great impact on the quality of life [3, 15]. Taken together, this illustrates that in this analysis men affected by PCa did not identify with the term “survivor” in order to reflect a cured disease state or to actively embrace their personal achievements in the fight against their disease but in order to express being burdened by the therapy and its long-term consequences. In such a context, men affected by PCa may consider themselves as wounded “survivor” of PCa and radical prostatectomy.

Research on how to incorporate a cancer experience in one’s identity has shown that some individuals choose to embrace actively engaging with their disease as part of their identity and that these individuals might benefit from such approach with better psychological well-being as well as disease coping [10, 19]. In the survivorship movements in the USA, an identification as a “cancer survivor” has been promoted as an exemplification of such active approach. But as being mentioned above without the proper context, the term might not feel as suitable to individuals affected by cancer that want to incorporate an active overcoming of their disease within their identity. In this survey, men were given the option to choose the more exaggerated term “cancer conqueror” to express such an outlook on their PCa experience. Men who chose “cancer conqueror” as self-description were less likely to report cancer-related distress or an ongoing therapy and were more likely to report higher quality of life as well as high benefit finding. These results suggest that a rather confident and active way of cancer-related self-perception in men affected by PCa might be a sign of a good psychological adaptation and that these men should be encouraged in embracing such an empowering approach. Another explanation for these results might be that a so far indistinct moderator such as an optimistic and extroverted personality might give rise to a rather confident and active way of self-description as well as lower distress and higher psychological well-being.

Only a minority of men in this analysis self-identified with the term “victim,” which stresses the seemingly pitiful fate and demanding therapy side effects of individuals diagnosed with and treated for cancer. Most studies on the subject have found that self-perception as a “cancer victim” is associated with psychological distress [8]. Interpretation of the data on men that self-identify as “victims” are limited as they only make up a small portion. Nevertheless, our data suggests that these men were more likely to be burdened with incontinence, high perceived disease severity, and anxiety, indicating the need for further psycho-oncological support.

The findings of this analysis have to be considered within certain limitations. Due to the cross-sectional design, causal assumptions on development of certain aspects of self-perception after radical prostatectomy should be further investigated in longitudinal studies. It is unclear whether certain psychological conditions after being affected by cancer might trigger identification with a certain cancer-related self-perception or whether actively embracing an empowering cancer-related identity might lead to increased psychological resilience and comfort. By only including men, who were primarily treated with radical prostatectomy, generalization towards all men affected by PCa is limited, and implications for other cancer types must be treated with caution. However, radical prostatectomy, which leads to sudden and potentially permanent changes in the lives of affected men, is the most common form of primary therapy for men with localized PCa. Therefore, our data on a large sample of nearly 1800 men represents a substantial portion of men affected by PCa, who often require continued medical assistance after primary therapy. All information on the functional outcome after radical prostatectomy are patient reported and therefore do not equal a clinical diagnosis. Nevertheless, assessment prior and post-surgery validates their clinical strength. Men were prompted to choose one of 5 terms that described them best with regard to their PCa experience. By applying forced choice, our data might represent self-perception after radical prostatectomy for PCa somewhat one-dimensionally. However, the different ways of self-perception were uniquely associated with distinctive clinical and psychological factors implying clinical relevant variance in the measured self-perception.

To conclude, most men with localized and effectively treated PCa perceive themselves in a neutral way, which represents their disease experience as something of the past such as “someone who has had cancer,” and it seems appropriate to address these men in such a way. Nevertheless, self-perception after treatment for PCa with radical prostatectomy can vary widely and is associated with distinct clinical and psychological characteristics that reflect the experienced severity of the disease, therapy side effects, and psychological well-being. Therefore, the assessment of cancer-related self-perception can give important insights when evaluating men affected by PCa who need assistance in coping with their disease.

## Data Availability

All available data are included into the manuscript.
